# MB0 and MBI Are Independent and Distinct Transactivation Domains in MYC that Are Essential for Transformation

**DOI:** 10.3390/genes8050134

**Published:** 2017-05-06

**Authors:** Qin Zhang, Kimberly West-Osterfield, Erick Spears, Zhaoliang Li, Alexander Panaccione, Stephen R. Hann

**Affiliations:** Department of Cell and Developmental Biology, Vanderbilt University, School of Medicine, 1121 21st Ave., Nashville, TN 37232, USA; qin.zhang@Vanderbilt.Edu (Q.Z.); kim.west@vanderbilt.edu (K.W.-O.); erick.spears@vanderbilt.edu (E.S.); zhaoliang.li@vanderbilt.edu (Z.L.); alex.panaccione@vanderbilt.edu (A.P.)

**Keywords:** MYC, transcription, transactivation, transformation, apoptosis

## Abstract

MYC is a transcription factor that is essential for cellular proliferation and development. Deregulation or overexpression of MYC occurs in a variety of human cancers. Ectopic expression of MYC causes hyperproliferation and transformation of cells in culture and tumorigenesis in several transgenic mouse models. Deregulation of MYC can also induce apoptosis through activation of p53 and/or ARF tumor suppressors as a safeguard to prevent tumorigenesis. MYC binds to thousands of genomic sites and regulates hundreds of target genes in a context-dependent fashion to mediate these diverse biological roles. The N-terminal region of MYC contains several conserved domains or MYC Boxes (MB), which influence the different MYC transcriptional and biological activities to varying degrees. However, the specific domains that mediate the ability of MYC to activate transcription remain ill defined. In this report, we have identified a new conserved transactivation domain (TAD), MB0, which is essential for MYC transactivation and target gene induction. We demonstrate that MB0 and MBI represent two distinct and independent TADs within the N-terminal 62 amino acids of MYC. In addition, both MB0 and MBI are essential for MYC transformation of primary fibroblasts in cooperation with activated RAS, while MB0 is necessary for efficient MYC-induced p53-independent apoptosis.

## 1. Introduction

c-Myc (MYC) is a member of a transcription factor family that includes MYCN, MYCL and MYCB and is essential for normal cellular proliferation, growth, metabolism, stem cell self-renewal and pluripotency [1]. MYC is the most ubiquitously expressed member of the family and deregulation of its activity drives tumorigenesis in up to 70% of human cancers [1,2]. Overexpression of MYC promotes hyperproliferation, transformation of cultured cells, tumorigenesis, angiogenesis, metastasis, genomic instability and metabolic reprogramming [1]. Apoptosis is induced by oncogenic MYC through both p53-dependent and independent mechanisms [3,4]. While non-transcriptional roles have been proposed for MYC [5,6], the transcriptional regulation of target genes likely mediates the majority of the diverse and numerous cellular processes controlled by MYC [7,8]. However, the mechanism(s) by which MYC induces or represses the transcription of these target genes is not well defined.

**Figure 1:**
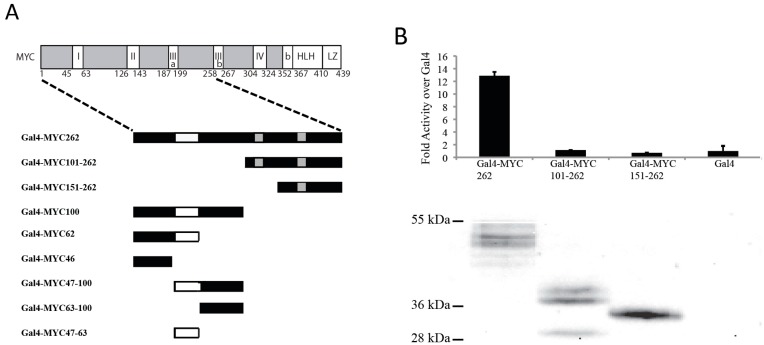

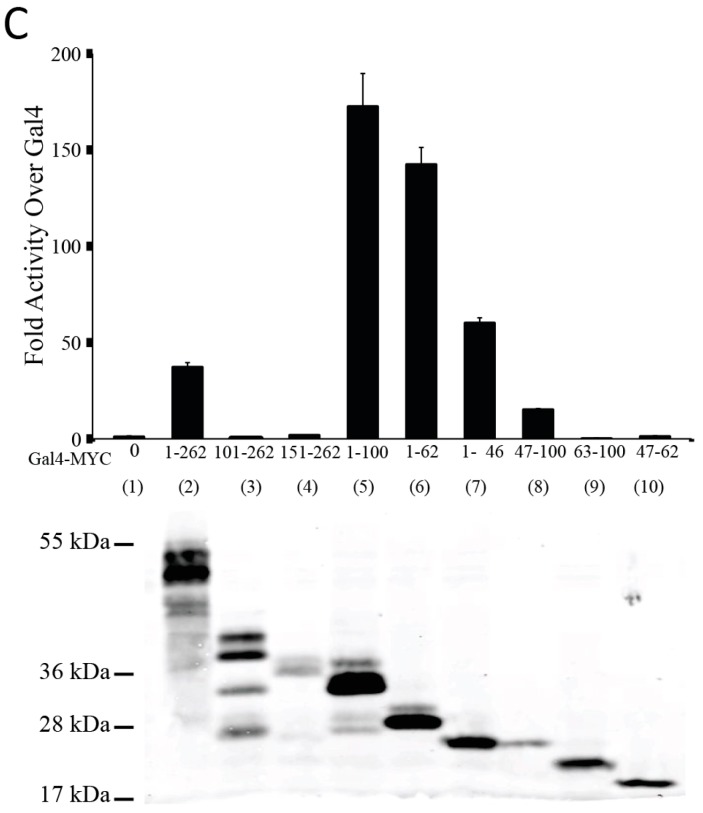
Identification of domains necessary for MYC-mediated transactivation. (**A**) Schematic of MYC protein structure showing MYC Boxes I through IV, the DNA binding domain (b), the helix–loop–helix (HLH) domain and the leucine zipper (LZ) domain. Dotted lines indicate the region of MYC used in the Gal4 fusions. The various Gal4-MYC fusion proteins used in the luciferase assays are shown below the schematic of the full-length structure; (**B**) The transactivation domain is within the N-terminal 100 amino acids of MYC. Gal4 assays were performed in Cos7 cells transfected with Gal4-MYC262, Gal4-MYC101-262, Gal4-MYC151-262 or empty Gal4 as described in the Materials and Methods. The bar graph represents transactivation normalized to Gal4-MYC protein levels. Gal4-MYC protein levels were determined by immunoblot (IB) analysis as described in the Materials and Methods; (**C**) Two regions within the N-terminal 62 amino acids of MYC retain transcriptional activity. Gal4 assays were performed and analyzed as described above using fragments within the N-terminal 100 amino acids of MYC compared to Gal4MYC100 and Gal4MYC262.

There are several regions of MYC, which are conserved between species and MYC family members that are essential for full transcriptional activity and biological functions. The C-terminal domain contains the basic (b) region, helix–loop–helix (HLH) region and leucine zipper (LZ) as shown in Figure 1A. The HLH–LZ region mediates heterodimerization with MAX and the basic region mediates binding of MYC/MAX to DNA at a canonical E-box MYC Site (EMS), CACGTG [9,10]. However, MYC/MAX has also been shown to bind to noncanonical sites [11,12]. In addition, there are conserved domains or MYC Boxes (MB) throughout the protein as shown in Figure 1A, which influence the ability of MYC to induce transformation and apoptosis [7,13].

The specific regions necessary for MYC to transactivate have not been clearly defined. To identify transactivation domains in transcription factors, investigators typically fuse specific protein regions to the yeast Gal4 DNA binding domain and assess activity using a reporter construct [14,15]. MYC is a potent transactivator in these assays, but is a relatively poor inducer of specific endogenous target genes and in transient promoter reporter assays [7]. Initial studies found that three arbitrarily chosen regions of the MYC N-terminus (1–43, 41–103 and 103–143) were all capable of activating transcription to varying degrees in Gal4 transactivation assays [16]. Based on these studies, the region containing the N-terminal 1–143 amino acids was termed the MYC TAD. In apparent disagreement with these results, the naturally occurring, alternative translational form MYC-S, which lacks the N-terminal 100 amino acids, cannot induce the majority of canonical target gene promoters, suggesting that MBII is insufficient to mediate transactivation [17,18,19]. In addition, deletion of MBII does not affect transactivation of most reporter constructs [20]. Further complicating the identification of the transactivation domain, MBI is not necessary for efficient transactivation and induction of some canonical target genes [16,21,22]. Another conserved domain, (NC1/MB0) has been identified within residues 1–43, but its role in the transcriptional activity of MYC in not clear [23]. Therefore, the relative contribution of the different domains for transactivation has not been resolved.

Although it is unclear whether the transactivation domain of MYC is restricted to the N-terminal 100 amino acids missing in MYC-S or whether MBI is an essential domain for transactivation, it is clear that the N-terminal 100 amino acids are critical for several MYC biological functions. MYC-S is deficient in inducing apoptosis and cell cycle entry from quiescence in mortal WI-38 human fibroblasts [18]. Unlike the full-length human MYC, human MYC-S also fails to induce apoptosis in *Drosophila* [24]. In contrast, MYC-S is capable of inducing hyperproliferation, apoptosis and soft agar growth of Rat1a cells, rescuing the slow growth phenotype of *MYC*^−/−^ Rat1 fibroblasts and rescuing the viability and growth of a lethal mutation of the *Drosophila myc* ortholog [24,25]. MYC-S also largely retains the ability to repress target genes [17,19]. Taken together, these studies suggest that the N-terminal 100 amino acids are not necessary for proliferation, transformation and apoptosis in immortalized cells or *Drosophila* development, but are essential for cell cycle entry and efficient apoptosis of primary cells.

In this report, we demonstrate that the N-terminal 62 amino acids of MYC mediate transactivation. In addition, a conserved domain from amino acids 10–32, MB0, is essential for MYC transactivation and induction of canonical MYC target genes. MBI is also necessary for efficient transactivation, independently of MB0. Loss of either MB0 or MBI has no effect on MYC-induced hyperproliferation of Rat1a cells, which is consistent with MYC-S studies, but both MB0 and MBI are necessary for cotransformation of primary rat embryonic fibroblast (REF) cells with activated RAS. In contrast, loss of MB0, but not MBI, inhibits the ability of MYC to efficiently induce p53-independent apoptosis. Therefore, with the identification of MB0 as a TAD, we demonstrate that there are two independent and distinct TADs in MYC.

## 2. Materials and Methods

### 2.1. Cell Culture, Transfection, and Retroviral Infection

*p53*^−/−^ MEFs and *p53*^−/−^/*ARF*^−/−^ double knockout (DKO) MEFs were cultured in DMEM with 10% charcoal striped calf serum (CS). Primary Fisher 344 rat embryonic fibroblasts (REF, Artis Optimus) were maintained in DMEM with 10% FBS. Rat1a and Cos-7 cells were maintained in DMEM with 10% CS. Cos-7 and REF cells were transfected using calcium phosphate precipitation with the indicated plasmids and subjected to analysis approximately 48 h after transfection. The *p53*^−/−^ MEFs, DKO MEFs and Rat1a cells stably expressing MYCER or deletion mutants were generated as described previously [26].

### 2.2. Plasmids and Expression Vectors

The MYC expression vectors pRcCMV-MYC and retroviral pBabe puro-MYCER have been previously described [26]. pCBS-MYC and pSP6-H-RasG12V were used for the MYC/RAS cotransformation assay. pFR-luc (firefly luciferase) was supplied by Stratagene. To generate Gal4-MYC fusion proteins, the pcDNA3 plasmid containing the coding sequence for the DNA binding domain of the yeast Gal4 transcription factor was used. Primers containing 5′ BamH1 and 3′ Xba1 restriction sites were used to amplify MYC fragments. The PCR fragments were digested and inserted into the 3′ end of the Gal4 coding sequence. The primers were as follows: MYCfwd: 5′-GGATCCCGATGCCCCTCAAGG-3′; MYC47fwd:5′-CCGGGATCCGTGAGGATATCTG-3′; MYC63fwd:5′-GCGGATCCCCCCGAGCCGCC-3′; MYC-Sfwd: 5′-GGATCCAGATGATGACCGAG-3′; MYC150 fwd: 5′-GGATCCTGGCCTCCTACCAGG-3′; MYC46rev:5′-TCCATCTAGATTAACTGGGCGCGG-3′; MYC62rev:5′-AGCGTCTAGATTAGGACAGGGGC-3′; MYC100rev:5′-GGTTCTAGATTACAGCTGATCGGCG-3′; MYC262rev:5′-GGTTTGCCTCTTCTCCACAGATCTAGATTAAATTTC-3′. To generate MYC deletion mutants, pcDNA3 Gal4-MYC100 was used as a template. Site directed mutagenesis was performed to generate the various deletions using the Site-Directed Mutagenesis kit (Stratagene) per the manufacturer’s instructions. The primers 5′-CTGCAGCCGCCCGCGCCCCCGAGCCGCCGCTC-3′ and 5′-GAGCGGCGGCTCGGGGGCGCGGGCGGCTGCAG-3′ were used to delete MBI; 5′-CAACGTGAACTTCACCAACAATTTCTATCACCAGCAACAG-3′ and 5′-CTGTTGCTGGTGATAGAAATTGTTGGTGAAGTTCACGTTG-3′ were used to delete MB0; 5′-CTGCGACGAGGAAGAGCTGCAGCCGCCCGCG-3′ and 5′-CGCGGGCGGCTGCAGCTCTTCCTCGTCGCAG-3′ were used to delete PolyQ; and 5′-CAACGTGAACTTCACCAACCTGCAGCCGCCCGCG-3′ and 5′-CGCGGGCGGCTGCAGGTTGGTGAAGTTCACGTTG-3′ were used to delete MB0 and PolyQ. All constructs were verified by DNA sequencing.

### 2.3. Reporter Assay

Cos-7 cells were seeded in 6-well plates to reach 80% confluency the next day and cells were transfected by calcium phosphate precipitation with various Gal4-MYC expression vectors and equal amount of pFR-Luciferase (Stratagene). pRL-TK (Renilla luciferase) was used as an internal control. Six hours post transfection the media was replaced by fresh culture media and transfected cells were incubated for at least 40 h. Two days post-transfection cells were lysed in Passive Lysis Buffer (Promega) and luciferase activity was assessed in triplicate for each sample using the BrightGlo Luciferase Assay kit (Promega) and Pharmigen’s LumiLight single tube luminometer. Results are expressed in fold difference over Gal4 activity and normalized to relative MYC protein expression using densitometry (Licor Odyssey).

### 2.4. Immunoblotting

Cell lysates were prepared in lysis buffer described in Qi, et al., 2004 [26]. Total protein concentration of lysates was assessed using the Bio-Rad *D_C_* Protein Assay system. Individual proteins were resolved by loading 10 µg of total protein per sample onto SDS-PAGE and subjected to immunoblot (IB) analysis using antibody raised against full length MYC or a monoclonal Gal4 antibody (Upstate). Blots were visualized using the Odyssey Infrared Imaging System (Licor). In order to resolve immunoreactive proteins with the Odyssey scanner, secondary antibodies conjugated to infrared spectra fluorophores were utilized. These secondary antibodies included goat anti-mouse AlexaFluor 680, donkey anti-sheep AlexaFluor 680 (Invitrogen) and donkey anti-rabbit IRDye 800 (Rockland).

### 2.5. MYC/RAS Cotransformation

Cotransformation of rat embryo fibroblasts (REFs) by MYC/RAS was performed as previously described [4]. REFs were plated at a density of 8 × 10^5^ cells/10 cm dish. The next day, cells were transfected by calcium phosphate precipitation with 12.5 µg pCßS-MYC2, ∆MB0, ∆MBI or empty vector plus an equal amount of pSP6-H-RasG12V. The media was removed and replaced with DMEM containing 4% FBS 24 h later and then every 3 days for the duration of the experiment. Two weeks after transfection, cells were stained with methylene blue and colonies were counted using BioRad’s ChemiDoc and Quantity One software. The numbers of colonies and the surface areas of visible colonies were tallied and averaged together. The experiments were repeated three times.

### 2.6. Proliferation and Apoptosis Assays

To assay proliferation, Rat1a cells expressing wild type MYC or the deletion mutants were seeded at a density of 2 × 10^4^ cells/12-well plate. Cells were fed every other day with fresh culture medium described above. To assay apoptosis, *p53*^−/−^ MEFs stably expressing MYCER or the indicated MYCER deletion mutants were seeded at 1 × 10^5^ cells/well in 6-well plates. The day after seeding, cells were shifted to media containing 1% CS and treated with 2 μM hydroxytamoxifen (OHT) daily. The number of attached cells (living cells) and floating cells (apoptotic cells) was determined in triplicate at the indicated times by counting with a hemacytometer. Apoptosis was confirmed by detection of caspase-3 activation using a specific antibody (Pab CM1; BD PharMingen). Each time course was representative of two different monoclonal cell lines.

### 2.7. Quantitative Real-Time RT-PCR (qRT-PCR)

DKO MEFs stably expressing MYCER, MYCER∆MB0, MYCER∆MBI or empty vector were plated at a density of 1 × 10^6^/10 cm dish. Forty hours post plating, cells were shifted to DMEM containing 0.1% charcoal stripped CS for 72 h followed by activation of MYC with 2 µM OHT for 12 h. Total RNA was isolated using the RNeasy Mini kit (Qiagen). Four µg of total RNA were treated with DNA-free DNAse (Ambion) and reverse transcribed using the iScript cDNA synthesis kit (Bio-Rad). qRT-PCR was performed using the Bio-Rad iCycler with iQ SYBR Green supermix (Bio-Rad). Relative measurement of gene expression was calculated using the standard curve method. Relative values compared to the actin level and to the 0 h time point were graphed as the mean ± SD from triplicate assays. Each assay is representative of at least two independent experiments. qPCR primers were as follows. Actin fwd: 5′-GCTGTGCTATGTTGCTCTAG-3′; rev: 5′-CGCTCGTTGCCAATAGTG-3′; hsp60 fwd: 5′-GGTGGCCTCCTTGCTAACTAC-3′; rev: 5′-CCCATTCCAGGGTCCTTCTCTT-3′; cdk4 fwd: 5′-TCACGCCTGTGGTGGTTAC-3′; rev: 5′-CGGGTGTTGCGTATGTAGAC-3′; mouse rcl sense: 5′-GGTTCCAGGTGTGGGACTACG-3′; antisense: 5′-GAAGATAAGCCTCAAAGTACCG-3′.

### 2.8. Data Analysis and Statistics

All data were collected from three independent experiments. Quantitative data are shown in graphic format indicating average values from all experiments with error bars showing variability ± standard deviation. All statistical analyses generated p-values using a single-tailed, pairwise Student’s *t*-test. Compared data sets were considered to be significantly different if the *p*-value generated was less than 0.05. 

## 3. Results

### 3.1. The N-Terminal 62 Amino Acids Represent the Transactivation Domain of MYC

We first examined the N-terminal 262 amino acids of MYC (Figure 1A, top), to determine which regions are necessary for transactivation. A series of N-terminal fragments of MYC was fused to the DNA binding domain of Gal4 (Figure 1A, bottom) and the various MYC-Gal4 proteins were transfected with a Gal4 luciferase reporter construct into Cos-7 cells. We first determined whether the region from 101–262 contained in MYC-S is able to transactivate. Figure 1B shows that both Gal4-MYC101–262 and Gal4-MYC151–262 were inactive, similar to the Gal4 empty vector, in contrast to the positive control Gal4-MYC262. The MYC-Gal4 proteins were expressed at similar levels as determined by immunoblot (IB) analysis (Figure 1B, bottom). This suggests that the region containing MBII and MBIIIa, which is retained in MYC-S, does not have the ability to transactivate. Therefore, the region that is responsible for the transcriptional activity of MYC is contained within the N-terminal 100 amino acids, which is lacking in MYC-S.

We next determined which region(s) within the N-terminal 100 amino acids is able to transactivate. Figure 1C shows that the Gal4-MYC62 amino acids had comparable activity to Gal4-MYC100, while Gal4-MYC 63–100 had no activity. This suggests that the TAD of MYC is restricted to the N-terminal 62 amino acids. The activity of the Gal4-MYC262 was significantly less than that of Gal4-MYC100 or Gal4-MYC62, suggesting that there is an inhibitory domain within amino acids 100–262. Interestingly, both Gal4-MYC 47–100, which includes the previously established TAD in MBI, and Gal4-MYC46 containing NC1/MB0 show activity, although not as high as Gal4-MYC62. This suggests that there are two independent TADs within the N-terminal 62 amino acids that contribute to the full activity of MYC.

### 3.2. Identification of a Conserved MYC Transactivation Domain

**Figure 2:**
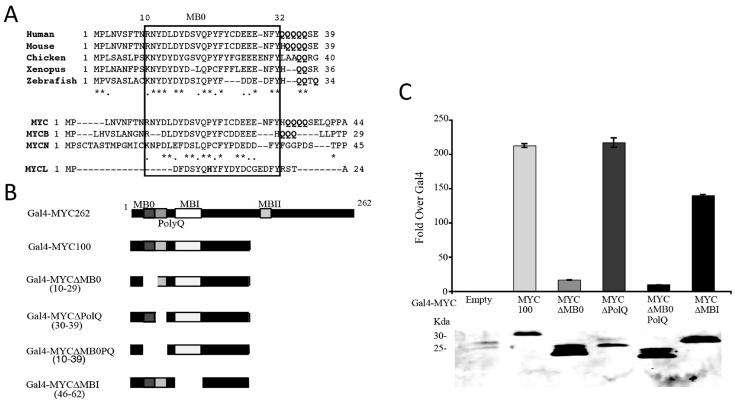
Identification of a novel conserved domain essential for MYC-mediated transactivation. (**A**) Sequence comparison of the N-terminal 45 amino acids of *MYC* from several different species and *MYC* family members. Stars identify exact amino acid matches and periods denote chemically similar amino acids. MYC Box 0 (MB0) is defined by the box containing amino acids 10–32 of human MYC. The regions of polyglutamine (Poly Q) amino acids are illustrated by boldface; (**B**) Schematic showing specific deletions of MB0, PolyQ region and MBI in Gal4MYC100 used in the Gal4 assays; (**C**) The MB0 domain is essential for MYC-mediated transactivation. Gal4 assays were performed and analyzed as described in Figure 1 using the various Gal4MYC100 deletion constructs described above.

The N-terminal 46 amino acids of MYC retained significant activity in comparison with the N-terminal 62 amino acid containing the MBI TAD. Conserved domains have previously been identified in the N-terminal 46 amino acid region, termed NC1 between amino acids 14–28 [23,27] or MB0 between 13–32 [28]. Amino acid sequence alignments comparing MYC from other species as well as from different family members revealed a region of high similarity between amino acids 10 and 32, similar to previously described NC1 and MB0 (Figure 2A). Further BLAST searches using this sequence revealed that MB0 is unique to MYC. Adjacent to MB0, there is a polyglutamine (PolyQ) tract in several of the different MYC sequences (Figure 2A). To determine the significance of the MB0 and PolyQ domains for MYC transactivation, Gal4-MYC100 fusion proteins were generated with deletions of these domains and of MBI for comparison (Figure 2B) and examined using the Gal4 transactivation assay. Figure 2C demonstrates that deletion of MB0 alone or combined with the PolyQ region significantly decreased transactivation compared to Gal4-MYC100, but deletion of the PolyQ region alone had no effect. This suggests that MB0, but not the adjacent PolyQ region, is essential for MYC transcriptional activity. Deletion of MBI also resulted in less activity, although not as dramatically as the MB0 deletion. Taken together, these data suggest that MB0 is the primary domain that mediates MYC transactivation, but that MBI is necessary for full activity.

### 3.3. MB0 Is Necessary for Endogenous Target Gene Induction

**Figure 3:**
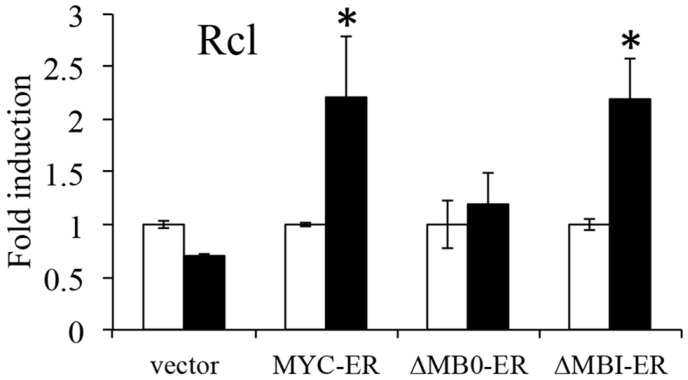

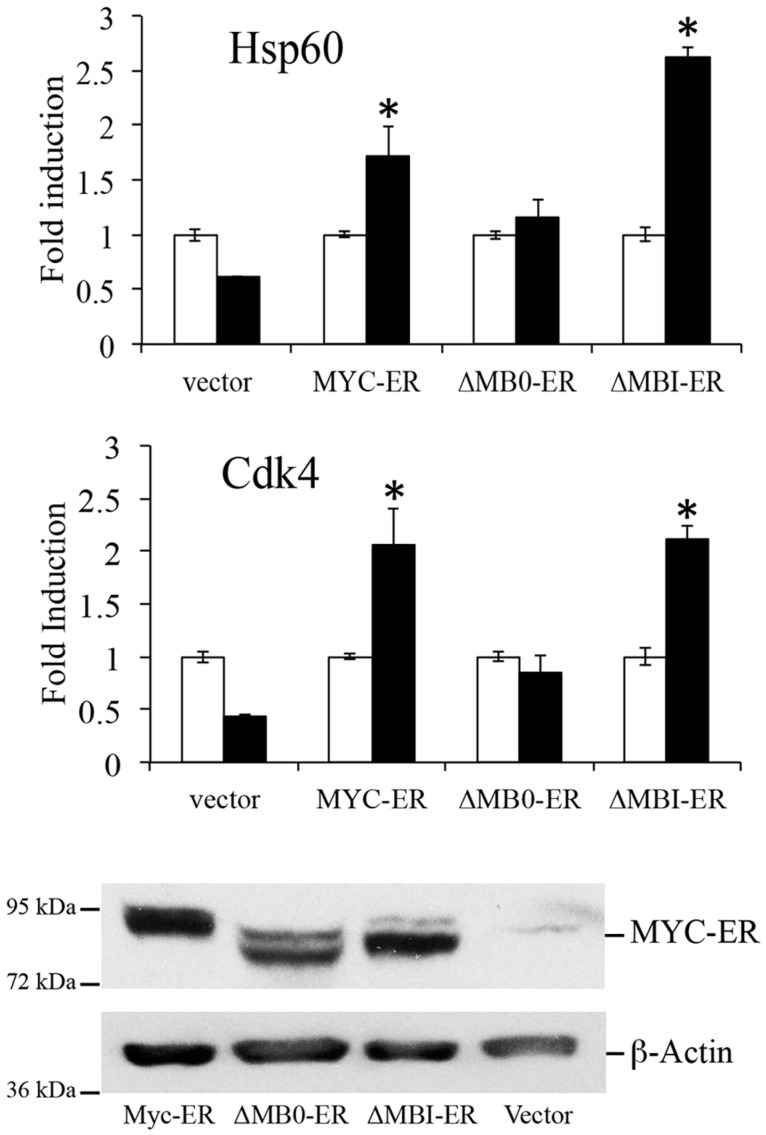
MB0 is essential for induction of MYC target genes. Stable *p53*^−/−^*/ARF*^−/−^ DKO MEFs expressing MYCER, MYCER∆MB0, MYCER∆MBI or empty vector were treated with 2 µM hydroxytamoxifen (OHT) for 0 or 12 h. Quantitative Real-Time RT-PCR (qRT-PCR) was performed as described in the Materials and Methods used to assess the levels of target genes *Rcl*, *Hsp60* and *Cdk4*. Results were normalized to ß-actin levels and shown as fold induction over levels at 0 h. Open bars indicate mRNA levels prior to OHT treatment, closed bars indicate mRNA levels after 12 h OHT treatment. Asterisks indicate statistically significant increases in mRNA after treatment with OHT as determined by Student’s *t*-test (*p* < 0.05, *n* = 3 per treatment group). MYCER protein expression of the various constructs was determined by IB analysis.

To determine the significance of MB0 for the ability of MYC to induce endogenous target gene upregulation, inducible MYC expression vectors were generated with deletions of MB0 and MBI. MYC, MYC∆MB0 and MYC∆MBI were fused to a modified domain of the estrogen receptor (ER), allowing activation in the presence of hydroxytamoxifen (OHT; [29,30]). We examined the ability of MYCER∆MB0 and MYCER∆MBI to induce several established canonical MYC target genes in *p53*^−/−^*/ARF*^−/−^ (DKO) MEFs. DKO MEFs were used to eliminate the inhibition of target gene expression by ARF as previously shown [26]. Several DKO MEF lines were isolated that expressed comparable levels of the different MYC proteins (Figure 3). The DKO MEFs were serum starved for 3 days to reduce endogenous MYC and then OHT was added to activate the MYCER proteins. Figure 3 shows that both MYCER and MYCER∆MBI induced the target genes *Rcl*, *Hsp60* and *Cdk4*, while MYCER∆MB0 was unable to induce any of these target genes. Therefore, these results suggest that the MB0 domain is essential for MYC target gene induction in this context.

### 3.4. MB0 and MBI Are Essential for Cotransformation of Primary Cells with Activated RAS

**Figure 4:**
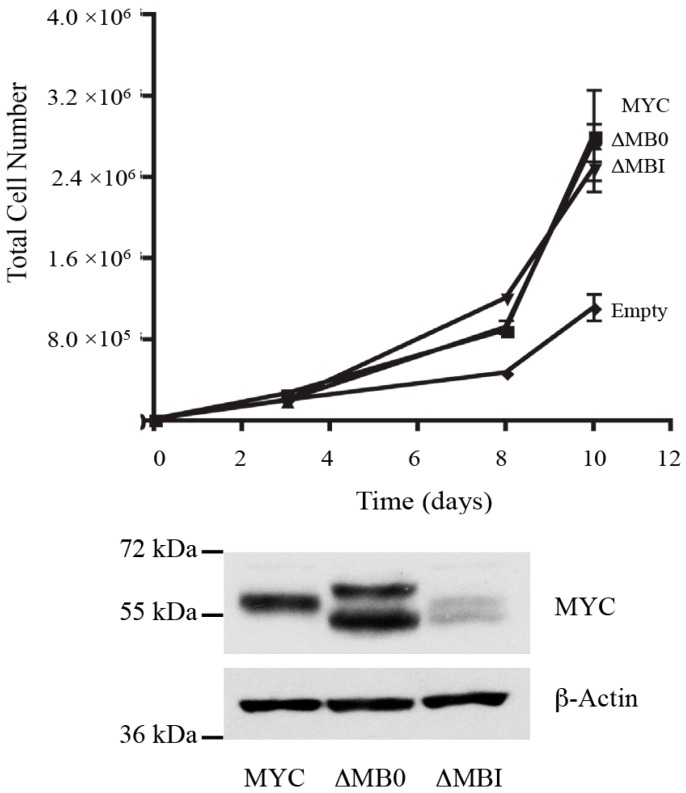
MB0 and MBI are dispensable for MYC-induced hyperproliferation of Rat1a fibroblasts. Immortalized polyclonal Rat1a cells stably expressing MYC, MYC∆MB0, MYC∆MBI or empty vectors were subjected to proliferation assays as described in the Materials and Methods. Expression of deletion mutants of MYC are visualized by IB analysis (below). Lower molecular weight bands in the doublets, ∆MB0 and ∆MBI lanes, indicate the exogenously expressed deletion mutants.

To examine the biological activity of MB0, we first assessed the ability of the MB0 and MBI deletion mutants to induce hyperproliferation of Rat1a cells compared to MYC. Based on previous MYC-S studies, deletions within the N-terminal 100 amino acids should retain the ability to cause hyperproliferation in these cells [25], unless the deletions created an inactive protein due to misfolding. Using Rat1a cells expressing similar amounts of the different proteins, both MYCΔMB0 and MYCΔMBI were comparable to full length MYC in causing hyperproliferation of Rat1a cells, confirming that the deletions did not generate nonfunctional proteins (Figure 4).

**Figure 5:**
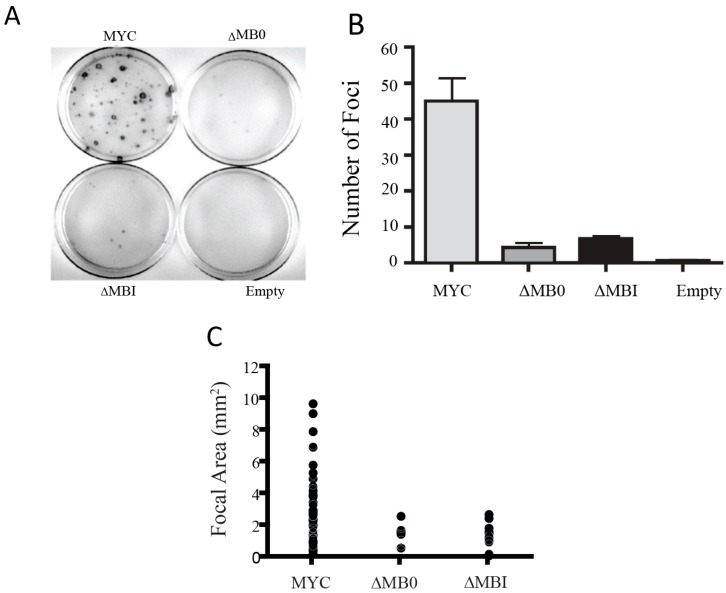
MB0 and MBI are both essential for MYC/RAS cotransformation in primary rat embryo fibroblasts. Low passage rat embryonic fibroblasts (REFs) were plated and cotransfected with H-Ras G12V and MYC, MYC∆MB0, MYC∆MBI or empty vector as described in the Materials and Methods. (**A**) Representative plates from each MYC cotransformation assay. The number of visible foci (**B**) and the sizes of individual foci (**C**) were also determined.

To determine the requirement of MB0 and MBI for the ability of MYC to transform primary rat embryo fibroblasts (REFs), we coexpressed MYC, MYC∆MB0 or MYC∆MBI with RAS. Coexpression of MYC with RAS resulted in numerous foci with a range of sizes (Figure 5). In contrast, coexpression of either MYC∆MB0 or MYC∆MBI with RAS generated relatively few foci and the few foci that did emerge were substantially smaller than those caused by MYC (Figure 5). Therefore, both MB0 and MBI are essential for the ability of MYC to efficiently cotransform REFs with RAS, suggesting that full transactivation mediated by both domains is essential for transforming primary cells.

### 3.5. MB0, but Not MBI, Is Necessary for the Efficient Induction of p53-Independent Apoptosis

**Figure 6:**
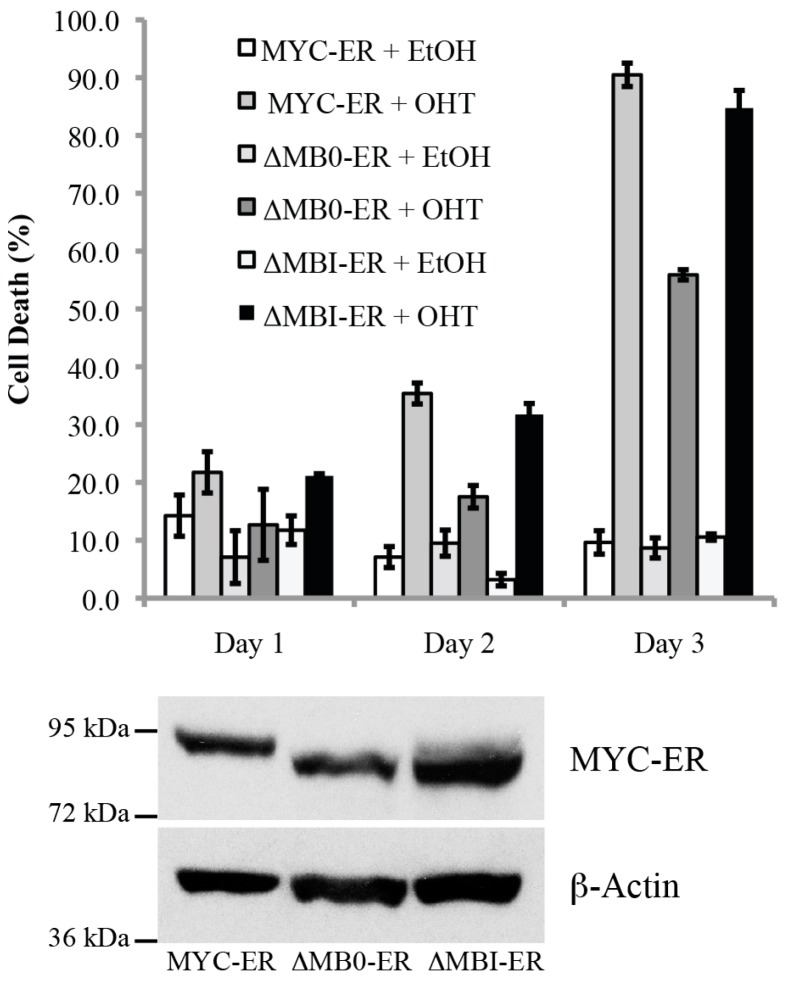
MB0, but not MBI, is essential for efficient MYC-induced p53-indendent apoptosis in MEFs. Stable *p53*^−/−^ MEFs expressing MYCER, MYCER∆MB0 or MYCER∆MBI were shifted to low serum and treated with 2 µM OHT or ethanol as described in the Materials and Methods. The percentage of dead cells was determined daily. All cultures treated with OHT showed statistically significant increases in cell death by Day 2 of treatment as determined by Student’s *t*-test (*p* < 0.05). MYCER protein expression of the various constructs was determined by IB analysis.

Previous MYC-S studies have also suggested that the N-terminal 100 amino acids are necessary for the ability of MYC to induce apoptosis efficiently in some cell types, including mortal WI-38 human fibroblasts [18]. To determine whether MB0 or MBI are necessary for the induction of p53-independent apoptosis, several *p53*^−/−^ MEF lines were established that expressed comparable levels of the inducible MYCER, MYCER∆MB0 and MYCER∆MBI proteins (Figure 6). We have previously shown that MYCER induces apoptosis in *p53^−/−^* MEFs, dependent on the endogenous tumor suppressor ARF [29,30]. Figure 6 shows that both MYCER and MYCER∆MBI initiated apoptosis by the second day and achieved apoptosis in approximately 90% of the cells by the third day following OHT activation. In contrast, MYCER∆MB0 showed little effect until the third day, when it induced apoptosis in approximately half the cells (Figure 6), significantly less than that induced by MYC-ER or ∆MB0-ER (Student’s *t*-test, *p* < 0.05). Therefore, these results suggest that the MB0 domain, but not MBI, is necessary for the full apoptotic activity of MYC in *p53*^−/−^ fibroblasts.

## 4. Discussion

We have identified a new conserved transactivation domain, MB0, near the N-terminus of MYC (amino acids 10–32). We demonstrate that MB0 and MBI represent two independent and distinct TADs within the N-terminal 62 amino acids of MYC. The earlier designation of the N-terminal 143 amino acids of MYC as the TAD was based on studies showing that several regions throughout the N-terminal 143 amino acids could transactivate [16]. The major difference between our results and the previous study is that we did not detect activity within amino acids 63–262, which includes MBII, and MBIIIa. Our results are supported by previous MYC-S studies [17,19]. In addition, MYCN-S is completely defective for RNA polymerase phosphorylation [19]. Taken together, these results suggest that the N-terminal 62 amino acids, which include MB0 and MBI, represent the MYC TAD, rather than the N-terminal 143 amino acids. The ability of MB0 to mediate transactivation appears to be critical for endogenous target gene induction, at least under conditions typically used to assess induction. Although MBI is a TAD, the loss of MBI did not affect induction of the selected canonical target genes. This may be due in part to the different conditions used to assess transactivation as compared to analysis of endogenous target gene induction. Since MYC target gene induction is known to be context dependent, the use of different conditions or cell types is likely the basis for the inconsistent results reported in the literature about the requirement for MBI in MYC target gene induction. This suggests that MBI-mediated target gene regulation is context and/or signal dependent. It is also likely that the requirement for MB0 and/or MBI is target gene dependent.

Sequence comparisons between MYC and MYCN and between the different species of MYC reveal that there are conserved amino acids within MB0 that may be essential for transactivation. The conserved amino acids in the sequence are basic-N-X-D-X-acidic-X-X-hydrophobic-Q-P-polar-F-X-X-(acidic)^3–4^-F-Y (Figure 2A). The MYCB sequence differs at the ends of MB0, lacking the N and the FY residues. MYCB is C-terminally truncated family member with extensive homology to the N-terminal 143 amino acids of MYC. Because of this homology, MYCB behaves as a dominant negative inhibitor of MYC transactivation [31]. MYCL has the least conserved MB0 sequence. While MBI and MBII are conserved in MYCL, it lacks several of the conserved amino acids in MB0, including the first five amino acids, the invariant proline at position twenty-two and the hydrophobic amino acid at position twenty. Compared to MYC, MYCL is a weak transactivator [32] and induces a significantly smaller number of target genes in microarray studies [33]. Thus, the differences in the MYCL MB0 may in part contribute to its reduced transcriptional activity.

Table 1 summarizes our results showing that MB0 is necessary for MYC/RAS cotransformation and efficient apoptosis in MEFs, but not Rat1a hyperproliferation. These data reinforce earlier studies with MYC-S, which suggest that transactivation is necessary for cell cycle entry and efficient apoptosis in primary cells [18,19,21]. The requirement for MB0-mediated transactivation for transformation is also supported by the observations that MYCL and a MYC protein lacking the N-terminal 22 amino acids (dN2) are weak transforming proteins [32,33]. Previous studies have also shown that MYCL and MYC-S are deficient in inducing apoptosis in *MYC*^−/−^ Rat1 fibroblasts, while MBI is dispensable [21], supporting our results showing that MB0 is necessary for efficient p53-independent apoptosis in MEFs. Surprisingly, MYCL and a MYCΔ22 (dN2) mutant are actually more efficient in promoting reprogramming of fibroblasts into pluripotent stem cells, suggesting that MB0 may be detrimental to this process [33]. Thus, the different MBs may contribute differentially to the diverse biological functions of MYC through regulation of distinct sets of target genes.

The mechanisms and cofactors that mediate MYC transactivation have not been clearly defined. While Myc-1–88 has an intrinsically disordered structure, there are transient secondary structures within residues 23–33 and 48–65, suggesting that MB0 and MBI represent dynamic interaction domains [34]. Since MB0 and MBI are independent and distinct TADs, it is likely that they regulate specific sets of target genes, which may be dependent on different context-dependent cofactors. We have previously shown that MYC cofactors, such as the tumor suppressor ARF and the ubiquitin E3 ligase SKP2, can differentially control MYC transcriptional activity [3,35], leading us to propose the Cofactor Switch Model [36,37]. Based on this idea, we suggest that distinct cofactors differentially bind to MB0 and MBI to mediate differential induction of target genes. Transactivation by MYC appears to be mediated, at least in part, by enhanced RNA polymerase II elongation through interaction with P-TEFb, which interacts with MBI [38], but it is unclear how this is regulated and what other cofactors may be involved in controlling MBI-mediated transactivation. Several proteins have been shown to bind to the N-terminal 41 amino acids of MYC that includes MB0. A component of the mediator complex, CDK8, binds to this region, but the functional significance of this interaction is unclear [38]. JPO2, which complexes with the transactivator p75, also binds to this region and potentiates MYC-mediated transformation [39,40]. In addition, TRRAP interaction with MBII, which is critical for MYC/RAS cotransformation, is partially dependent on this region [4]. Recently, Pin1 binding to MBI has been shown to be dependent on MB0 [28]; however, the role of Pin1 in MYC transcriptional activity and biological function is controversial [41].

Although MBII is not a TAD, based on standard transactivation assays, transactivation mediated by MB0 and MBI is likely dependent on MBII for endogenous target gene expression due to its critical role in histone modification and chromatin remodeling. However, since MYC-S does induce a small set of target genes independently of MB0 or MBI, MBII may mediate target gene upregulation through other direct or indirect mechanisms, such as derepression. There is also the possibility that MBII may mediate transactivation under specific conditions, perhaps dependent on MBI or MB0. MBII is also critical for MYC-mediated repression of target genes [7]. MBIIIa is essential for both maximal induction and repression of MYC target genes and for transformation [22]. Therefore, the N-terminal half of MYC, which includes MB0, MBI, MBII and MBIIIa may be better designated as the Transcriptional Regulatory Domain (TRD) that mediates several different transcriptional mechanisms to distinguish it from the TAD. Since transactivation mediated by MB0 and MBI appears to be dispensable for inducing hyperproliferation, apoptosis and soft agar growth of Rat1a cells, rescuing the slow growth phenotype of *MYC*^−/−^ Rat1 fibroblasts and rescuing the viability and growth of a lethal mutation of the *Drosophila myc* ortholog, other activities mediated within the TRD, such as repression and epigenetic modifications, likely mediate these MYC functions [17,24,25]. Understanding which domains, mechanisms and interacting proteins are involved in MYC functions in different cells will allow a focused approach to controlling MYC activity for therapeutic intervention.

## 5. Conclusions

We have identified a new conserved transactivation domain, MB0, near the N-terminus of MYC. MB0 and MBI represent two independent and distinct TADs within the N-terminal 62 amino acids of MYC. Loss of either MB0 or MBI has no effect on MYC-induced hyperproliferation of immortalized Rat1a cells, which is consistent with MYC-S studies, but both MB0 and MBI are necessary for cotransformation of primary cells with activated RAS. In addition, loss of MB0, but not MBI, inhibits the ability of MYC to efficiently induce p53-independent apoptosis. Unlike MBI and MBII, MB0 is not conserved in MYCL, which is a weak transactivating and transforming protein and is deficient in inducing apoptosis in MYC^−/−^ Rat1 fibroblasts compared to MYC. We posit that MB0 and MBI induce distinct sets of target genes mediated by different cofactors in a context-dependent fashion. These target genes may independently control specific MYC biological functions or may need to synergize for other functions, such as transformation and tumorigenesis.

## Figures and Tables

**Table 1:** Summary of Results from Functional Assays Using MYC Transactivation Domain Deletions.

Functional Assay	Reference Figure	MYC	ΔMB0	ΔMBI
Gal4 Reporter	Figure 2	++	-	+
Endogenous Target Genes	Figure 3	+	-	+
Hyperproliferation	Figure 4	+	+	+
MYC/RAS Cotransformation	Figure 5	+	-	-
p53-Independent Apoptosis	Figure 6	++	+	++
